# Supplementary material for: Molecular Dynamics and MM-PBSA Analysis of the SARS-CoV-2 Gamma Variant in Complex with the hACE-2 Receptor
Source: Molecules. 2022 Apr 6;27(7):2370. doi: 10.3390/molecules27072370 (PMC9000566; doi:10.3390/molecules27072370)
Supplement: Supplementary file 1 [file molecules-27-02370-s001.zip › molecules-1564901-supplementary.pdf]

Supplementary Materials

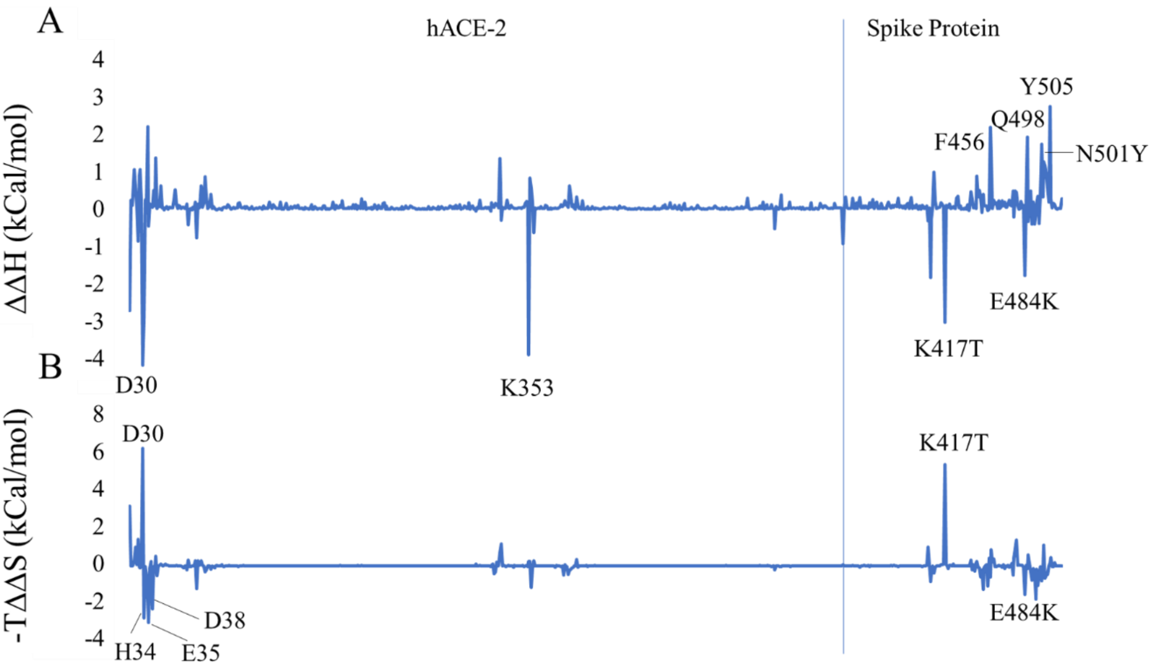

Figure S1. MM-PBSA and Interaction entropy residue specific contribution. $\Delta\Delta G_{total}$ contribution by residue. (**A**) $\Delta\Delta H$ for the wild type complex and gamma variant complex obtained with MM-PBSA technique. (**B**) $-T\Delta\Delta S$ for the wild type complex and gamma variant complex obtained by computing the interaction entropy.

Table S1. Cristalographic complexes of the six SARS-CoV-2 variants of concern.

PDB ID	Variant	Resolution [Å]	R-Free
7ekf [1]	B.1.1.7 (alpha)	2.85	0.224
7ekg [1]	B.1.351 (beta)	2.63	0.248
7ekc [1]	P.1 (gamma)	2.80	0.225
6m0j [2]	Wuhan-Hu-1 (wild type)	2.45	0.227
7wbq [3]	B.1.617.2 (delta)	3.34	0.226
7wbp [3]	B.1.1.529 (omicron)	3.00	0.204

1. Han, P.; Su, C.; Zhang, Y.; Bai, C.; Zheng, A.; Qiao, C.; Wang, Q.; Niu, S.; Chen, Q.; Zhang, Y.; et al. Molecular Insights into Receptor Binding of Recent Emerging SARS-CoV-2 Variants. *Nat. Commun.* **2021**, *12*, doi:10.1038/S41467-021-26401-W.

2. Lan, J.; Ge, J.; Yu, J.; Shan, S.; Zhou, H.; Fan, S.; Zhang, Q.; Shi, X.; Wang, Q.; Zhang, L.; et al. Structure of the SARS-CoV-2 Spike Receptor-Binding Domain Bound to the ACE2 Receptor. *Nature* **2020**, *581*, 215–220, doi:10.1038/s41586-020-2180-5.

3. Han, P.; Li, L.; Liu, S.; Wang, Q.; Zhang, D.; Xu, Z.; Han, P.; Li, X.; Peng, Q.; Su, C.; et al. Receptor Binding and Complex Structures of Human ACE2 to Spike RBD from Omicron and Delta SARS-CoV-2. *Cell* **2022**, *185*, 630-640.e10, doi:10.1016/J.CELL.2022.01.001.
